# Ocular manifestation in a patient with IgG4 related disease

**DOI:** 10.22088/cjim.13.1.127

**Published:** 2022

**Authors:** Samaneh Azizimanesh, Fatema Rizvi, Hamed Zainaldain, Samira Alesaeidi

**Affiliations:** 1 Research Center for Rheumatologic Diseases, Amir-Alam Hospital, Tehran University of Medical Sciences, Tehran, Iran; 2 Research Center for Immunodeficiencies, Children’s Medical Center, Tehran University of Medical Sciences, Tehran, Iran

**Keywords:** IgG4-related disease, IgG4-related orbitopathy, Visual acuity, Proptosis.

## Abstract

**Background:**

IgG4-related disease is a newly recognized fibroinflammatory disease presenting with multiple features including mass forming lesion; a dense lymphoplasmacytic infiltrate; a characteristic histopathological appearance and often elevated serum of IgG4. This disease can potentially affect any organ and interestingly, the affected organs share common histopathological features including a dense lymphoplasmacytic infiltrate rich in IgG4-positive plasma cells, storiform fibrosis, and mild to moderate eosinophilia

**Case Presentation:**

A 45-year-old man presented complaining of proptosis and gradual decrease in visual acuity of right eye. He had undergone many work-ups but without any definitive diagnosis. Through a combination of clinical and para-clinical investigations, the diagnosis of IgG4-RD was established. 693 mg/dL). Aggressive treatment (pulse of cyclophosphamide and pulse of corticosteroid) was started hoping to save the patient’s vision. Two weeks following the treatment, there was improvement with his visual acuity and proptosis.

**Conclusion:**

In any patient with chronic tumor like lesions and pseudotumors without the evidence of malignancy, we should think of IgG4-related disease. In this circumstance, biopsy may lead us to the definitive diagnosis. Early diagnosis and treatment of IgG4-RD may inhibit further irreversible organ damages.

IgG4-related disease (IgG4-RD) is relatively a new fibroinflammatory disease that can involve a variety of organs and tissues such as the hepatobiliary ducts, salivary glands, periorbital tissues, and lacrimal glands (1). This disease can potentially affect any organ and interestingly, the affected organs share common histopathological features including a dense lymphoplasmacytic infiltrate rich in IgG4-positive plasma cells, storiform fibrosis, and mild to moderate eosinophilia (1). It is characterized by tumefactive lesions, obliterative phlebitis, and a rise in serum IgG4 concentrations(2). The diagnosis is often challenging as none of the clinical, laboratory and histopathological findings are pathognomonic of this disease, and the diagnosis largely rely on the coexistence of all. Delay in diagnosis and treatment could have sometimes irreversible impact on the affected organ. We are going to introduce a patient with diagnosis of IgG4-RD with delay in diagnosis and treatment who suffered from the consequences of the disease as massive proptosis and decrease in visual acuity.

## Case presentation

**Figure 1:**
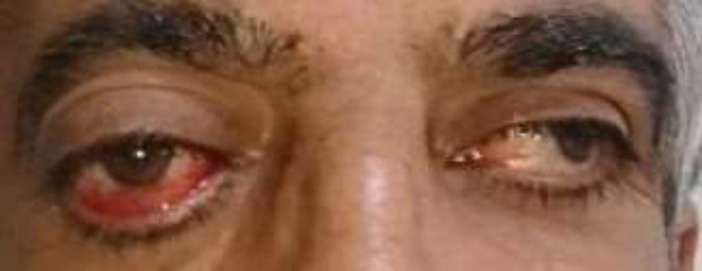
This picture is shot before the initiation of the treatment. Notice the right eye proptosis

A 45-year-old man referred to our Rheumatology clinic for further evaluation of proptosis, which started about 8 years ago. He had a gradual bilateral decrease of vision that worsened during the last 18 months. Physical examination revealed a proptosis of 33 mm and 26 mm in the right and left eyes, respectively (figure 1). 

**Figure2:**
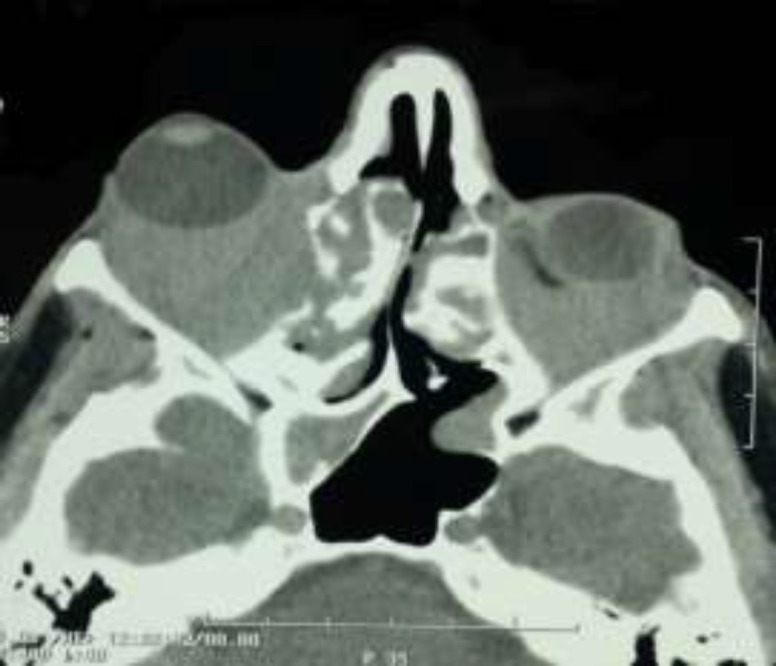
CT of PNS and orbits, showed evidence of bilateral retro-orbital enhancing masses notably in the right orbit with intraconal and extraconal extension encasement of optic nerve, and extension into the orbital apex and superior and inferior orbital

There was a mild conjunctivitis in both eyes and the visual acuity (best corrected visual acuity) of the right eye was recorded as hand motion recognition at 50 cm and that of the left eye was normal (9/10). He had a mild cataract in both eyes. On further eye examination, the anterior segments of both eyes and the posterior segment of left eye were normal, but the posterior segment of right eye had grade 2+ disc paleness. Marcus gun examination was positive. Fundoscopy of the right eye showed blurred disc margin and mild engorgement of veins around the disc. The rest of physical examinations were unremarkable. There were no skin or mucosal involvement, sinusitis, otitis, neurological or respiratory manifestations. Laboratory investigations yielded the following results: erythrocyte sedimentation rate (ESR) was 11 mm/h, white blood cells (WBC): 13000 /ul, hemoglobin: 13.7 gr/dl, platelet count: 404000 /ul, urea: 31 mg/dl, serum creatinine (Cr): 1 mg/dl, aspartate aminotransferase (AST): 10 U/L, alanine transaminase (ALT):12 U/L, alkaline phosphatase (ALP): 187 U/L, INR:1 and total bilirubin: 0.2mg/dl. Urinalysis was normal without any cellular cast, dysmorphic RBCs or proteinuria. Echocardiography indicated normal left ventricle size with ejection fraction (EF) of 55%, normal left ventricular systolic function, moderate aortic regurgitation, and a bicuspid aortic valve. In computed tomography of paranasal sinuses and orbits, there was an evidence of bilateral retro-orbital enhancing masses notably in the right orbit with intraconal and extraconal extension encasement of optic nerve, and extension into the orbital apex and superior and inferior orbital fissures (figure 2). There was an extension into frontal and ethmoidal air cells and also inferior extension into nasal cavity and both maxillary sinuses with widening of ostiomeatal complex (OMC) bilaterally. There were significant proptosis and extension toward cavernous sinus at the right side.

The patient underwent four different ocular biopsies. In June 2014, the patient had microscopic anaplastic lymphoma kinase (ALK) positive inflammatory myofibroblastic tumor, and underwent 17 sessions of radiotherapy, but the clinical symptoms did not resolve. The last pathology study revealed chronic inflammatory pseudotumor with marked fibrosis and IgG4 positive plasma cells about 50/ high power field. The results of immunohistochemistry staining (IHC) study was as follows: IgG4 positive lymphoid cells 50/ high power field, CD138 positive plasma cells, CD3 positive T cells (80%), CD20 positive B cells (20%), smooth muscle antibody (SMA) positive spindle cells, negative ALK and his serum IgG4 level was 693 mg/dl. 

**Figure 3:**
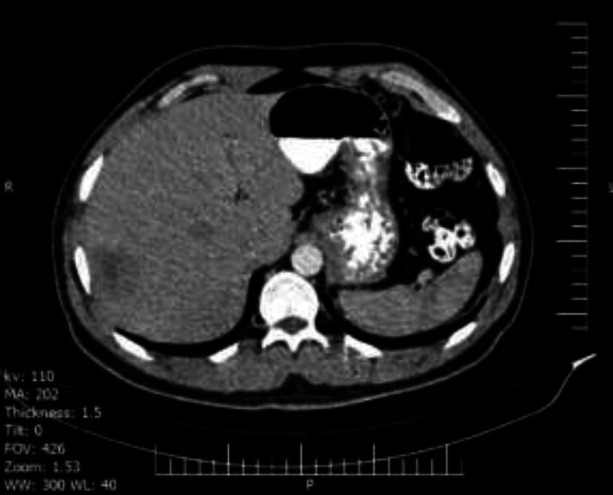
Abdominal CT revealed multiple bilateral hepatic mass lesions with the largest size being (41*32 mm) at segment 7, and 28*2 (3)7 mm at segment 3

As this disease can affect any organ and due to a complaint of mild abdominal pain by the patient, an abdominal CT scan was performed. The computed tomography scan results revealed multiple bilateral hepatic mass lesions with the largest size being (41*32 mm) at segment 7, and 28*2 (3)7 mm at segment 3 (Figure 3). The masses were non-specific and there was a high recommendation for pathologic evaluation to rule out metastatic lesions, lymphoma or other possible lesions. The patient accepted liver biopsy, and hemangioma was reported by pathology assessment.

**Figure 4:**
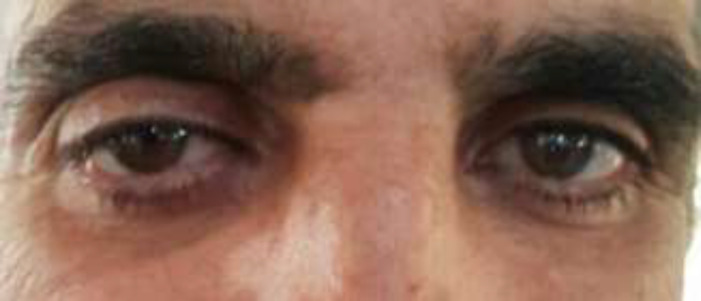
The patient after the treatment, the proptosis improved significantly and the visual acuity of right eye improved from hand motion to finger count at 1-meter

When the patient referred to us, the diagnosis of IgG4-related disease was established based on the clinical and histopathological findings including the result of IHC revealed the presence of IgG4 positive plasma cells, the ratio of IgG4>40% and serum IgG4 level>130 mg/dL (693 mg/dL). Aggressive treatment (pulse of cyclophosphamide and pulse of corticosteroid) started with methylprednisolone 1gram/ day for 3 consecutive days. We observed a significant reduction of symptoms as the proptosis decreased to 26mm and 17.5 mm in the right and the left eyes, respectively (figure 4). 

The visual acuity of right eye improved from hand motion to finger count at 1 meter. After 4 pulses of 1-gram cyclophosphamide/ month, his visual acuity improved to 3/10. The patient required a prolonged treatment with glucocorticoid, however, even though it was expensive, azathioprine was prescribed as a glucocorticoid-sparing therapy. The author previously reported a similar case of IgG4-RD with ocular manifestation of proptosis and decrease in visual acuity that showed a dramatic response to early aggressive treatment with corticosteroid and cyclophosphamide (4).

## Discussion

Involvement of head and neck is one of the most common presentations of IgG4-RD (5). Patients presented with dacro-cystitis and other head and neck disorders involving the extraocular muscles, sclera, cavernous sinuses, choroid and orbital soft tissue of the orbit (6). Differential diagnosis can be made based on the clinical presentation and the involved organs. If IgG4-RD involves pancreas (autoimmune pancreatitis type1) we should take into account other causes including biliary, drug induced or alcoholic pancreatitis and adenocarcinoma of pancreas. If it involves the salivary gland, Sjogren syndrome should be considered. Differentiation between these two diseases is based on the clinical features (eye/ mouth dryness) and serological findings (anti SSA/Ro – anti SSB/La). Differentiating between primary sclerosing cholangitis and cholangiocarcinoma with IgG4-related sclerosing cholangitis (IgG4-SC) is quite challenging, but it can be done with the help of imaging studies and serologic findings. Sarcoidosis can be differentiated from IgG4-RD based on chest CT findings, IgG4 level of broncho-alveolar lavage and IgG4 level of serum (3).

There are also some diseases that exhibit positive and elevated IgG4 cells in biopsy, for example inflammatory conditions such as primary sclerosing cholangitis (PSC), rheumatic arthritis (RA), inflammatory bowel disease (IBD), anti-neutrophil cytoplasmic antibody (ANCA)- associated vasculitis etc., but none of them shows the clinical and compatible histopathological features of IgG4-RD.

Lymphomas especially low- grade B-cell lymphoma must be ruled out when IgG4-RD is thought to be the diagnosis. IgG4 positive plasma cells can be seen in cancerous tissues as well, especially pancreatobilliary cancers, however, lack of other typical histopathological aspects of IgG4-RD, like storiform fibrosis or obliterative phlebitis make the diagnosis simple (7). Idiopathic orbital inflammation (IOI) is an inflammatory, nonmalignant and non-infectious disease that can affect any site in the orbit. The diagnosis of IOI is made by the exclusion of secondary diseases that may involve orbit. However, nowadays with the improvement in diagnostic capabilities, many patients who were previously labelled as IOI are now diagnosed as IgG4-related orbital disease (IgG4-ROD) (8). 

Various rheumatologic diseases can affect the orbital cavity including Wegener’s granulomatosis, giant cell arthritis, systemic lupus erythematosus (SLE), dermatomyositis and rheumatoid arthritis (RA) (8). Other causes of orbital inflammatory disease include infections, neoplasms and systemic inflammatory conditions as mentioned above (9). In this case, due to diagnostic delay as well as delay in treatment, the patient presented with complications such as massive proptosis and decrease of visual acuity. The primary pathology from the patient`s optical mass biopsy reported chronic inflammatory pseudotumor (benign lymphoma). Progression of the disease and worsening of proptosis and visual acuity of the patient probably are due to limited knowledge about the prevalence, manifestations and progression of IgG4-RD at 7-8 years ago. Unfortunately, this led to the deterioration in the visual acuity of the patient (hand motion at 50 cm). Eventually, in the last biopsy specimen from the patient`s retro-orbital mass, the pathologic sample was evaluated for IgG4 staining and the serum level of IgG4, as well as the ratio of IgG4/IgG, was evaluated too. 

The diagnosis of IgG4-RD was established based on the following findings; the presence of IgG4 positive plasma cells, the ratio of IgG4-RD >40%, and serum IgG4 level > 130 mg/dl. Early aggressive treatment with pulse cyclophosphamide and pulse corticosteroids) was started hoping to save the vision of the patient. Two weeks following the aggressive treatment, there was an improvement in the visual acuity of the patient to finger count at 1 meter, and after 4 pulses of cyclophosphamide, the visual acuity improved to 3/10. In this case, due to the dramatic response to pulse cyclophosphamide, we recommend cyclophosphamide pulse therapy to be used in the management of life threatening, organ involvement of the disease.

During hospitalization, due to the fact that IgG4-RD is a lymphoproliferative, multi-organ disease, we evaluated the patient for other organ involvement. In the abdominal CT scan, several hepatic masses were noticed, and biopsies were taken from most of the mass. Pathological evaluation of the biopsy specimens revealed hemangioma. MRI hepatic protocol was also performed for some of the masses in which the pathologic evaluation was not clear, and hemangiomas were reported too. In articles that were reviewed, the importance of the pathologic evidence of liver mass was emphasized, however there was no relationship reported between the incidence of hepatic hemangioma and IgG4-RD.

IgG4-related hepatobiliary disease (IgG4-HDs) is a part of fibroinflammatory multi-organ involvement of IgG4-RD, including IgG4-hapatopathy and IgG4-related sclerosing cholangitis. They can present as bile duct stenosis, to distinguish them from PSC and other hepatic malignancies (6). IgG4- HDs may be asymptomatic and are diagnosed accidentally during cross-sectional abdominal imaging for other reasons (6). There was no pancreatic involvement; Pancreas of the patient had normal size and shape. IgG4-RD is a benign disease that responds very well to treatment with corticosteroids, but discontinuation of the treatment may cause a relapse as in the case of our patient (10). He had been using a single dose corticosteroid per month for the last four years prescribed by his previous healthcare physician and showed slight improvement, but he discontinued the treatment, and suffered from relapse of the disease with manifestation of severe proptosis and significant decrease in visual acuity.

## Ethical Approval

All procedures performed in the study involving human participant were in accordance with the ethical standards of the institutional research committee and with the ethical standards.

## Informed Consent

Informed consent was obtained from the patient in the study. Written permission from the patient was taken for publication of this work with his photo.

## Conflict of Interest:

None of the authors have any conflict of interest.
